# Supplementary material for: Seasonal vertical migration of large polar copepods reinterpreted as a dispersal mechanism throughout the water column
Source: Commun Earth Environ. 2025 Jun 4;6(1):431. doi: 10.1038/s43247-025-02389-9 (PMC12137130; doi:10.1038/s43247-025-02389-9)
Supplement: Supplementary file 2 — Supplementary Information [file 43247_2025_2389_MOESM2_ESM.docx]

|  | **AF** | **AF** | **AF** | **CV** | **CV** | **CV** | **Eggs** | **Nauplii** |
| --- | --- | --- | --- | --- | --- | --- | --- | --- |
|  | Nov-Mar  2019/2020  (n = 14)  CAO | Jul  2020  (n = 3)  Fram St | Aug-Sept  2020  (n = 9)  CAO | Nov-Mar  2019/2020  (n = 14)  CAO | Jul  2020  (n = 3)  Fram St | Aug-Sept  2019/2020  (n = 9)  CAO | Nov-Mar  2019/2020  (n = 14)  CAO | Nov-Mar  2019/2020  (n = 14)  CAO |
| *Ind m^-2^* |  |  |  |  |  |  |  |  |
| **0-50 m** | 16±10 | 491±251 | 154±188 | 6±6 | 161±46 | 118±148 | 266±414 | 24±42 |
| **50-200 m** | 115±39 | 9±10 | 111±55 | 27±16 | 9±3 | 30±12 | 521±381 | 151±144 |
| **200-500 m** | 62±24 | 5±6 | 77±37 | 34±22 | 6±5 | 37±16 | 805±346 | 388±489 |
| **500-1000 m** | 113±54 | 15±6 | 115±56 | 151±66 | 9±3 | 91±55 | 768±216 | 868±785 |
| **1000-2000 m** | 152±89 | 29±13 | 181±144 | 264±172 | 42±18 | 304±318 | 685±240 | 493±602 |
| *mg C m^-2^* |  |  |  |  |  |  |  |  |
| **0-50 m** | 43±27 | 944±482 | 345±422 | 7±7 | 100±29 | 112±140 |  |  |
| **50-200 m** | 312±106 | 17±19 | 249±123 | 33±20 | 6±2 | 28±11 |  |  |
| **200-500 m** | 168±65 | 10±12 | 173±83 | 41±27 | 4±3 | 35±15 |  |  |
| **500-1000 m** | 306±146 | 29±12 | 258±126 | 184±80 | 6±2 | 86±52 |  |  |
| **1000-2000 m** | 412±241 | 56±25 | 406±323 | 322±210 | 26±11 | 288±301 |  |  |
| *%* |  |  |  |  |  |  |  |  |
| **0-50 m** | 3.7±2.8 | 87.4±7.5 | 20.1±20.6 | 1.3±2.0 | 71.3±2.4 | 18.0±14.1 | 8.0±11.1 | 1.5±2.5 |
| **50-200 m** | 27.2±12.4 | 2.2±3.1 | 16.6±7.5 | 7.5±7.7 | 4.1±1.5 | 8.7±9.2 | 16.7±9.4 | 9.9±12.0 |
| **200-500 m** | 13.7±5.5 | 0.7±0.7 | 13.5±6.1 | 9.1±8.6 | 2.7±2.8 | 8.3±4.6 | 27.1±10.5 | 18.2±20.5 |
| **500-1000 m** | 23.8±7.7 | 3.4±2.3 | 21.8±13.0 | 31.3±12.6 | 4.0±0.1 | 20.8±13.5 | 25.5±5.9 | 45.3±23 |
| **1000-2000 m** | 31.5±13.4 | 6.2±3.6 | 28.0±13.1 | 50.8±17.3 | 18.0±4.1 | 44.2±22.4 | 22.6±7.7 | 25.1+21.5 |

**Table S1:** Results of MultiNet samples from the MOSAiC expedition showing *C. hyperboreus* AF and CV abundances (Ind m^-2^), carbon biomass (mg C m^-2^) and relative distribution (%) (± SD) across five depth strata between 2000-0 m, in the Fram Strait (Jul 2020) and the CAO, ≥85°N (Nov. 2019 – Mar. 2020, Aug.-Sept. 2020). Copepod eggs and *Calanus* nauplii were not identified to species level.

The carbon content was 1.9, 2.2 and 2.7 mg C AF^-1^, and 0.6, 0.9 and 1.2 mg C CV^-1^ in Jul, Aug-Sept and Nov-Mar, respectively.

**Table S2:** Summary of results from the biochemical analysis of *C. hyperboreus* AF and CV sampled in the surface ocean (50-0 m, 200-50 m) *versus* deep ocean (1000-500 m, 2000-1000 m) during Aug.-Sept. 2020 in the CAO, ≥85°N (MOSAiC expedition). The presented data are the median values of 8-12 replicate measurements, with bold *p*-values indicating significant differences between those median values (Mann-Whitney U Test).

| **Median values** | *C. hyperboreus* - **AF** | | | | *C. hyperboreus* - **CV** | | | |
| --- | --- | --- | --- | --- | --- | --- | --- | --- |
|  | Surface (n=10) | Depth  (n=12) | *W*-value | *p*-value | Surface (n=8) | Depth (n=12) | *W*-value | *p*-value |
| **Dry mass** (mg ind^-1^) | 3.96 | 3.95 | 120 | 0.767 | 1.71 | 1.95 | 68 | 0.232 |
| **Prosome length** (mm) | 6.81 | 6.76 | 74 | 0.832 | 5.05 | 5.13 | 34 | **0.021** |
| **Lipid** (% dry mass) | 43.67 | 44.00 | 115 | 1.000 | 38.28 | 40.83 | 72 | 0.375 |
| **Wax ester** (% lipid) | 86.86 | 87.27 | 105 | 0.531 | 86.48 | 87.85 | 54 | **0.023** |
| **C/N** | 8.10 | 7.76 | 125 | 0.531 | 7.01 | 7.46 | 64 | 0.132 |
| **δ^13^C** | -27.82 | -26.42 | 64 | **0.001** | -28.23 | -26.99 | 58 | **0.049** |
| **δ^15^N** | 7.71 | 7.08 | 131 | 0.307 | 7.83 | 7.11 | 115 | **0.019** |
| **Fatty acids** (% TFA) |  |  |  |  |  |  |  |  |
| 14:0 | 4.24 | 4.36 | 90 | 0.106 | 3.71 | 4.29 | 52 | **0.015** |
| 16:0 | 4.38 | 3.92 | 160 | **0.003** | 3.78 | 3.14 | 130 | **0.001** |
| 16:1(n-7) | 31.65 | 30.58 | 139 | 0.121 | 27.30 | 22.83 | 105 | 0.114 |
| 18:1(n-9) | 3.82 | 3.38 | 157 | **0.006** | 4.46 | 3.42 | 114 | **0.023** |
| 18:1(n-7) | 1.89 | 1.78 | 133 | 0.249 | 1.60 | 1.46 | 99 | 0.263 |
| 18:4(n-3) | 2.00 | 0.97 | 158 | **0.005** | 5.18 | 2.56 | 119 | **0.008** |
| 20:5(n-3) | 9.80 | 9.98 | 117 | 0.921 | 12.88 | 11.49 | 108 | **0.07** |
| 22:6(n-3) | 6.20 | 5.20 | 163 | **0.002** | 9.27 | 6.03 | 123 | **0.003** |
| 20:1 | 14.22 | 16.80 | 70 | **0.003** | 9.98 | 16.49 | 40 | **0.001** |
| 22:1 | 10.36 | 12.57 | 81 | **0.027** | 7.83 | 15.04 | 38 | **0.001** |
| ∑ 20,22:1 FA | 24.58 | 29.59 | 74 | **0.008** | 17.80 | 31.10 | 39 | **0.001** |
| **Fatty acid ratios** |  |  |  |  |  |  |  |  |
| 16:1(n-7)/18:4(n-3) | 15.40 | 31.29 | 74 | **0.008** | 5.35 | 9.05 | 61 | 0.083 |
| 18:1(n-7)/18:1(n-9) | 2.01 | 1.81 | 144 | 0.06 | 2.81 | 2.25 | 107 | 0.083 |
| 16:4(n-1)/18:4(n-3) | 0.27 | 0.54 | 81 | **0.027** | 0.21 | 0.24 | 72 | 0.375 |
| 20:5(n-3)/18:4(n-3) | 4.94 | 9.92 | 68 | **0.002** | 2.54 | 4.49 | 57 | **0.041** |
| **Fatty alcohol** (% TFAlc) |  |  |  |  |  |  |  |  |
| 14:0 | 3.77 | 3.28 | 145 | 0.052 | 6.40 | 5.13 | 113 | **0.028** |
| 16:0 | 9.61 | 8.16 | 158 | **0.005** | 16.77 | 11.39 | 113 | **0.028** |
| 16:1 | 3.18 | 2.19 | 162 | **0.002** | 6.84 | 3.79 | 108 | 0.07 |
| 20:1 | 31.32 | 32.90 | 92 | 0.138 | 26.49 | 28.23 | 66 | 0.18 |
| 22:1 | 48.88 | 52.20 | 81 | **0.027** | 40.10 | 49.47 | 50 | **0.01** |
| ∑ 20,22:1 | 81.16 | 84.24 | 68 | **0.002** | 66.51 | 76.92 | 54 | **0.023** |
| **Phytosterols** (% TPS) |  |  |  |  |  |  |  |  |
| Brassicasterol | 31.29 | 29.35 | 121 | 0.717 | 32.25 | 35.85 | 69 | 0.263 |
| Chalinasterol | 30.29 | 25.57 | 125 | 0.531 | 33.86 | 26.19 | 107 | 0.083 |
| Sitosterol | 9.13 | 8.01 | 142 | 0.081 | 6.84 | 5.69 | 100 | 0.232 |
| Campersterol | 32.80 | 37.53 | 87 | 0.07 | 26.91 | 25.45 | 82 | 0.908 |
| **Polar lipids** (% PL) |  |  |  |  |  |  |  |  |
| 18:4(n-3) | 0.44 | 0.00 | 140 | 0.106 | 0.58 | 0.00 | 114 | **0.023** |
| 20:5(n-3) | 22.70 | 21.93 | 132 | 0.277 | 20.94 | 18.97 | 122 | **0.004** |
| 22:6(n-3) | 52.61 | 53.81 | 94 | 0.176 | 54.19 | 56.65 | 67 | 0.203 |
| ∑ PUFA | 75.65 | 74.64 | 115 | 1.000 | 77.15 | 75.50 | 95 | 0.418 |
| **Neutral lipids** (% NL) |  |  |  |  |  |  |  |  |
| 18:4(n-3) | 2.14 | 0.00 | 158.5 | **0.005** | 6.29 | 2.75 | 119 | **0.008** |
| 20:5(n-3) | 11.51 | 10.96 | 114 | 0.974 | 16.06 | 13.78 | 108 | 0.07 |
| 22:6(n-3) | 5.80 | 4.00 | 154 | **0.011** | 11.44 | 6.47 | 119 | 0.008 |
| ∑ PUFA | 20.14 | 15.47 | 152 | **0.016** | 34.11 | 24.49 | 121 | 0.005 |
| **δ^13^C-FA** |  |  |  |  |  |  |  |  |
| 14:0 | -30.90 | -30.23 | 87 | 0.07 | -31.73 | -31.94 | 89 | 0.728 |
| 16:0 | -28.91 | -28.09 | 94 | 0.176 | -29.57 | -29.09 | 68 | 0.232 |
| 16:1 | -26.63 | -27.54 | 133 | 0.249 | -27.85 | -27.84 | 87 | 0.847 |
| 18:1 | -30.42 | -30.75 | 120 | 0.767 | -30.82 | -30.55 | 75 | 0.512 |
| 18:4(n-3) | -36.72 | -33.43 | 44 | **0.008** | -35.21 | -29.83 | 46 | **0.004** |
| 20:5(n-3) | -34.19 | -32.46 | 84 | **0.044** | -33.36 | -30.39 | 43 | **0.002** |
| 22:6(n-3) | -32.17 | -30.03 | 81 | **0.027** | -32.25 | -28.96 | 49 | **0.008** |
| PUFA | -34.27 | -31.88 | 628 | **0.001** | -33.35 | -29.93 | 408 | **0.0001** |
| 20:1 | -31.80 | -31.44 | 98 | 0.277 | -31.03 | -29.48 | 67 | 0.203 |
| 22:1 | -28.68 | -28.07 | 91 | 0.121 | -27.28 | -26.32 | 78 | 0.671 |
| **δ^13^C-FAlc** |  |  |  |  |  |  |  |  |
| 14:0 | -27.54 | -26.87 | 87 | 0.07 | -28.18 | -27.82 | 64 | 0.132 |
| 16:0 | -24.68 | -24.01 | 95 | 0.199 | -28.26 | -27.02 | 61 | 0.083 |
| 16:1 | -27.01 | -22.00 | 77 | **0.013** | -31.04 | -26.24 | 49 | **0.008** |
| 20:1 | -31.46 | -29.78 | 62 | **0.001** | -30.80 | -29.85 | 61 | 0.083 |
| 22:1 | -28.33 | -26.73 | 88 | 0.081 | -26.02 | -26.44 | 85 | 0.969 |
| **ATP-gain (**molecules) |  |  |  |  |  |  |  |  |
| PL-FA | 123.49 | 123.76 | 79 | **0.019** | 124.40 | 124.61 | 67 | 0.203 |
| NL-FA | 117.66 | 118.68 | 84 | **0.044** | 117.96 | 121.20 | 45 | **0.003** |
| NL-FAlc | 133.44 | 134.59 | 76 | **0.011** | 127.71 | 131.41 | 56 | **0.034** |

**Abbreviations:** FA- fatty acid, FAlc – fatty alcohol, TFA – total FA, TFAlc – total Alc, TPS – total phytosterols, PL- polar lipids, NL – neutral lipids

The number of ATP molecules that can be gained via beta-oxidation were calculated based

on the average number of carbon atoms and the average number of double bonds within the total FA or FAlc pool (<http://amazingbiotech.in/biochemical-calculator/>)

**Table S3:** Results of MultiNet samples from the MOSAiC expedition showing the average abundances (ind m^-2^) of potential predators of *C. hyperboreus* lifestages (eggs, nauplii, copepodites, adults) across five depth strata between 2000-0 m, in Nov. 2019 – Mar. 2020 (CAO, ≥85°N), July 2020 (Fram Strait) and Aug.-Sept. 2020 (CAO, ≥85°N). *Metridia longa, Paraeuchaeta* spp*.,* Ostracoda*, Scaphocalanus magnus, Themisto abyssorum,* Chaetognatha*,* Cnidaria*, Cyclocaris guilelmi, Hymenodora glacialis*

| **Date** | **Strata** | ***Metridia***  ***longa***  **AF** | ***Paraeuch.***  **spp.**  **AF** | **Ostracoda** | **Scaphoc. magnus**  **AF** | ***Themisto***  ***abyssorum*** | **Chaetogn** | **Cnidaria** | ***Cycloc.***  ***guilelmi*** | ***Hymend.***  ***glacialis*** |
| --- | --- | --- | --- | --- | --- | --- | --- | --- | --- | --- |
|  |  | **ind m^-3^** | **ind m^-3^** | **ind m^-3^** | **ind m^-3^** | **ind m^-3^** | **ind m^-3^** | **ind m^-3^** | **ind m^-3^** | **ind m^-3^** |
| 14/11/2019 | 50-0 m | 13.636 | 0.000 | 11.818 | 0.545 | 0.091 | 0.182 | 0.091 | 0.000 | 0.000 |
| 14/11/2019 | 200-50 m | 1.000 | 0.028 | 3.306 | 0.083 | 0.306 | 0.972 | 0.750 | 0.000 | 0.000 |
| 14/11/2019 | 500-200 m | 0.028 | 0.000 | 0.806 | 0.000 | 0.083 | 3.722 | 0.917 | 0.000 | 0.000 |
| 14/11/2019 | 1000-500 m | 0.025 | 0.000 | 0.644 | 0.017 | 0.000 | 3.831 | 0.475 | 0.028 | 0.000 |
| 14/11/2019 | 2000-1000 m | 0.015 | 0.000 | 0.239 | 0.000 | 0.000 | 0.008 | 0.317 | 0.004 | 0.004 |
|  |  |  |  |  |  |  |  |  |  |  |
| 28/11/2019 | 50-0 m | 9.231 | 0.231 | 12.077 | 0.231 | 0.077 | 1.154 | 0.385 | 0.000 | 0.000 |
| 28/11/2019 | 200-50 m | 0.098 | 0.024 | 1.000 | 0.000 | 0.073 | 0.878 | 0.415 | 0.000 | 0.000 |
| 28/11/2019 | 500-200 m | 0.048 | 0.000 | 0.500 | 0.000 | 0.000 | 3.238 | 0.286 | 0.000 | 0.000 |
| 28/11/2019 | 1000-500 m | 0.020 | 0.000 | 0.324 | 0.000 | 0.000 | 0.703 | 0.182 | 0.007 | 0.000 |
| 28/11/2019 | 2000-1000 m | 0.015 | 0.000 | 0.240 | 0.000 | 0.000 | 0.012 | 0.240 | 0.003 | 0.000 |
|  |  |  |  |  |  |  |  |  |  |  |
| 5/12/2019 | 50-0 m | 7.467 | 0.000 | 8.200 | 0.133 | 0.133 | 1.933 | 0.067 | 0.000 | 0.000 |
| 5/12/2019 | 200-50 m | 0.651 | 0.000 | 2.907 | 0.116 | 0.116 | 2.186 | 0.651 | 0.000 | 0.000 |
| 5/12/2019 | 500-200 m | 0.023 | 0.000 | 0.651 | 0.000 | 0.023 | 3.767 | 0.674 | 0.000 | 0.000 |
| 5/12/2019 | 1000-500 m | 0.007 | 0.000 | 0.293 | 0.007 | 0.000 | 0.776 | 0.259 | 0.000 | 0.000 |
| 5/12/2019 | 2000-1000 m | 0.054 | 0.000 | 0.302 | 0.000 | 0.003 | 0.044 | 0.319 | 0.003 | 0.000 |
|  |  |  |  |  |  |  |  |  |  |  |
| 21/12/2019 | 50-0 m | 7.833 | 0.083 | 8.833 | 0.083 | 0.333 | 0.167 | 0.000 | 0.000 | 0.000 |
| 21/12/2019 | 200-50 m | 0.541 | 0.054 | 2.703 | 0.162 | 0.297 | 0.351 | 0.432 | 0.000 | 0.000 |
| 21/12/2019 | 500-200 m | 0.056 | 0.000 | 0.514 | 0.014 | 0.111 | 1.319 | 0.556 | 0.000 | 0.000 |
| 21/12/2019 | 1000-500 m | 0.008 | 0.000 | 0.285 | 0.000 | 0.000 | 0.350 | 0.333 | 0.008 | 0.000 |
| 21/12/2019 | 2000-1000 m | 0.020 | 0.000 | 0.150 | 0.000 | 0.000 | 0.004 | 0.259 | 0.000 | 0.000 |
|  |  |  |  |  |  |  |  |  |  |  |
| 26/12/2019 | 50-0 m | 7.733 | 0.133 | 5.733 | 0.133 | 0.067 | 0.600 | 0.067 | 0.000 | 0.000 |
| 26/12/2019 | 200-50 m | 0.789 | 0.000 | 3.158 | 0.158 | 0.184 | 0.921 | 0.447 | 0.000 | 0.000 |
| 26/12/2019 | 500-200 m | 0.111 | 0.000 | 0.514 | 0.014 | 0.139 | 3.417 | 0.306 | 0.000 | 0.014 |
| 26/12/2019 | 1000-500 m | 0.023 | 0.000 | 0.336 | 0.000 | 0.000 | 0.183 | 0.336 | 0.008 | 0.000 |
| 26/12/2019 | 2000-1000 m | 0.077 | 0.000 | 0.046 | 0.000 | 0.000 | 0.008 | 0.176 | 0.004 | 0.000 |
|  |  |  |  |  |  |  |  |  |  |  |
| 2/1/2020 | 50-0 m | 0.000 | 0.077 | 7.154 | 0.231 | 0.000 | 0.308 | 0.154 | 0.000 | 0.000 |
| 2/1/2020 | 200-50 m | 0.357 | 0.000 | 1.976 | 0.095 | 0.024 | 0.476 | 0.286 | 0.000 | 0.000 |
| 2/1/2020 | 500-200 m | 0.012 | 0.000 | 0.346 | 0.012 | 0.037 | 0.630 | 0.284 | 0.000 | 0.000 |
| 2/1/2020 | 1000-500 m | 0.007 | 0.000 | 0.306 | 0.000 | 0.007 | 0.366 | 0.328 | 0.007 | 0.007 |
| 2/1/2020 | 2000-1000 m | 0.042 | 0.000 | 0.340 | 0.000 | 0.000 | 0.019 | 0.205 | 0.000 | 0.000 |
|  |  |  |  |  |  |  |  |  |  |  |
| 7/1/2020 | 50-0 m | 8.500 | 0.000 | 7.833 | 0.000 | 0.083 | 0.083 | 0.000 | 0.000 | 0.000 |
| 7/1/2020 | 200-50 m | 1.500 | 0.028 | 4.306 | 0.083 | 0.056 | 0.361 | 0.722 | 0.000 | 0.000 |
| 7/1/2020 | 500-200 m | 0.029 | 0.014 | 0.500 | 0.014 | 0.014 | 1.143 | 0.771 | 0.057 | 0.000 |
| 7/1/2020 | 1000-500 m | 0.017 | 0.008 | 0.085 | 0.000 | 0.000 | 0.169 | 0.331 | 0.017 | 0.000 |
| 7/1/2020 | 2000-1000 m | 0.012 | 0.000 | 0.116 | 0.000 | 0.000 | 0.012 | 0.145 | 0.008 | 0.000 |
|  |  |  |  |  |  |  |  |  |  |  |
| 14/1/2020 | 50-0 m | 6.000 | 0.154 | 7.615 | 0.077 | 0.077 | 0.462 | 0.231 | 0.000 | 0.000 |
| 14/1/2020 | 200-50 m | 0.390 | 0.000 | 2.293 | 0.098 | 0.073 | 2.732 | 0.390 | 0.000 | 0.000 |
| 14/1/2020 | 500-200 m | 0.013 | 0.000 | 0.494 | 0.038 | 0.013 | 1.127 | 0.468 | 0.000 | 0.000 |
| 14/1/2020 | 1000-500 m | 0.000 | 0.000 | 0.294 | 0.000 | 0.008 | 1.476 | 0.190 | 0.000 | 0.000 |
| 14/1/2020 | 2000-1000 m | 0.026 | 0.000 | 0.124 | 0.008 | 0.000 | 0.026 | 0.282 | 0.008 | 0.000 |
|  |  |  |  |  |  |  |  |  |  |  |
| 22/1/2020 | 50-0 m | 2.231 | 0.000 | 4.000 | 0.000 | 0.077 | 0.000 | 0.000 | 0.000 | 0.000 |
| 22/1/2020 | 200-50 m | 0.462 | 0.026 | 1.410 | 0.128 | 0.026 | 0.744 | 0.718 | 0.000 | 0.000 |
| 22/1/2020 | 500-200 m | 0.038 | 0.013 | 0.291 | 0.000 | 0.038 | 0.367 | 0.329 | 0.000 | 0.000 |
| 22/1/2020 | 1000-500 m | 0.000 | 0.000 | 0.273 | 0.008 | 0.008 | 0.689 | 0.417 | 0.008 | 0.015 |
| 22/1/2020 | 2000-1000 m | 0.023 | 0.000 | 0.116 | 0.000 | 0.000 | 0.016 | 0.349 | 0.004 | 0.000 |
|  |  |  |  |  |  |  |  |  |  |  |
| 29/1/2020 | 50-0 m | 8.615 | 0.077 | 12.692 | 0.308 | 0.385 | 0.231 | 0.308 | 0.000 | 0.000 |
| 29/1/2020 | 200-50 m | 0.553 | 0.000 | 3.500 | 0.158 | 0.237 | 2.263 | 0.895 | 0.000 | 0.000 |
| 29/1/2020 | 500-200 m | 0.123 | 0.000 | 0.727 | 0.027 | 0.000 | 1.814 | 0.604 | 0.000 | 0.000 |
| 29/1/2020 | 1000-500 m | 0.008 | 0.000 | 0.420 | 0.000 | 0.000 | 0.351 | 0.450 | 0.000 | 0.008 |
| 29/1/2020 | 2000-1000 m | 0.049 | 0.000 | 0.355 | 0.000 | 0.000 | 0.012 | 0.339 | 0.012 | 0.000 |
|  |  |  |  |  |  |  |  |  |  |  |
| 4/2/2020 | 50-0 m | 5.000 | 0.143 | 6.143 | 0.000 | 0.071 | 0.000 | 0.000 | 0.000 | 0.000 |
| 4/2/2020 | 200-50 m | 0.692 | 0.026 | 2.333 | 0.128 | 0.077 | 1.231 | 0.872 | 0.000 | 0.000 |
| 4/2/2020 | 500-200 m | 0.014 | 0.000 | 0.417 | 0.028 | 0.014 | 2.111 | 0.625 | 0.000 | 0.000 |
| 4/2/2020 | 1000-500 m | 0.009 | 0.000 | 0.293 | 0.000 | 0.009 | 0.319 | 0.241 | 0.000 | 0.000 |
| 4/2/2020 | 2000-1000 m | 0.009 | 0.000 | 0.225 | 0.004 | 0.004 | 0.013 | 0.247 | 0.000 | 0.000 |
|  |  |  |  |  |  |  |  |  |  |  |
| 18/2/2020 | 50-0 m | 6.133 | 0.000 | 7.400 | 0.200 | 0.000 | 0.867 | 0.200 | 0.000 | 0.000 |
| 18/2/2020 | 200-50 m | 1.045 | 0.000 | 1.636 | 0.159 | 0.091 | 1.273 | 0.705 | 0.000 | 0.000 |
| 18/2/2020 | 500-200 m | 0.000 | 0.000 | 0.138 | 0.000 | 0.011 | 0.492 | 0.253 | 0.000 | 0.000 |
| 18/2/2020 | 1000-500 m | 0.000 | 0.000 | 0.125 | 0.000 | 0.000 | 0.778 | 0.333 | 0.000 | 0.000 |
| 18/2/2020 | 2000-1000 m | 0.003 | 0.000 | 0.182 | 0.000 | 0.000 | 0.038 | 0.213 | 0.000 | 0.000 |
|  |  |  |  |  |  |  |  |  |  |  |
| 7/3/2020 | 50-0 m | 0.037 | 0.000 | 0.167 | 0.000 | 0.000 | 0.093 | 0.056 | 0.000 | 0.000 |
| 7/3/2020 | 200-50 m | 2.667 | 0.039 | 2.667 | 0.078 | 0.098 | 0.333 | 0.176 | 0.000 | 0.000 |
| 7/3/2020 | 500-200 m | 0.025 | 0.013 | 0.278 | 0.013 | 0.013 | 0.519 | 1.000 | 0.000 | 0.013 |
| 7/3/2020 | 1000-500 m | 0.008 | 0.008 | 0.132 | 0.000 | 0.000 | 0.093 | 0.504 | 0.008 | 0.008 |
| 7/3/2020 | 2000-1000 m | 0.000 | 0.000 | 0.158 | 0.004 | 0.000 | 0.016 | 0.265 | 0.004 | 0.000 |
|  |  |  |  |  |  |  |  |  |  |  |
| 12/3/2020 | 50-0 m | 0.357 | 0.000 | 0.857 | 0.000 | 0.000 | 0.143 | 0.214 | 0.000 | 0.000 |
| 12/3/2020 | 200-50 m | 1.564 | 0.077 | 2.000 | 0.128 | 0.000 | 1.077 | 0.615 | 0.000 | 0.000 |
| 12/3/2020 | 500-200 m | 0.000 | 0.000 | 0.185 | 0.022 | 0.000 | 0.315 | 0.554 | 0.000 | 0.000 |
| 12/3/2020 | 1000-500 m | 0.370 | 0.000 | 0.191 | 0.000 | 0.000 | 0.006 | 0.309 | 0.011 | 0.000 |
| 12/3/2020 | 2000-1000 m | 0.003 | 0.000 | 0.069 | 0.000 | 0.000 | 0.021 | 0.152 | 0.006 | 0.003 |
|  |  |  |  |  |  |  |  |  |  |  |
| 17/8/2020 | 50-0 m | 3.214 | 0.071 | 2.857 | 0.000 | 1.000 | 0.500 | 0.214 | 0.000 | 0.000 |
| 17/8/2020 | 200-50 m | 0.707 | 0.024 | 6.098 | 0.122 | 0.024 | 1.610 | 0.366 | 0.000 | 0.000 |
| 17/8/2020 | 500-200 m | 0.065 | 0.013 | 0.377 | 0.000 | 0.117 | 1.558 | 0.779 | 0.000 | 0.000 |
| 17/8/2020 | 1000-500 m | 0.030 | 0.007 | 0.193 | 0.000 | 0.000 | 0.148 | 0.304 | 0.015 | 0.000 |
| 17/8/2020 | 2000-1000 m | 0.000 | 0.000 | 0.083 | 0.004 | 0.000 | 0.022 | 0.338 | 0.000 | 0.000 |
|  |  |  |  |  |  |  |  |  |  |  |
| 18/8/2020 | 50-0 m | 2.750 | 0.000 | 2.833 | 0.000 | 0.000 | 0.583 | 0.083 | 0.000 | 0.000 |
| 18/8/2020 | 200-50 m | 1.692 | 0.000 | 5.923 | 0.154 | 0.051 | 0.615 | 0.359 | 0.000 | 0.000 |
| 18/8/2020 | 500-200 m | 0.000 | 0.013 | 0.461 | 0.013 | 0.013 | 1.382 | 0.632 | 0.000 | 0.000 |
| 18/8/2020 | 1000-500 m | 0.008 | 0.000 | 0.214 | 0.000 | 0.000 | 0.855 | 0.664 | 0.008 | 0.000 |
| 18/8/2020 | 2000-1000 m | 0.000 | 0.000 | 0.072 | 0.017 | 0.000 | 0.051 | 0.212 | 0.004 | 0.000 |
|  |  |  |  |  |  |  |  |  |  |  |
| 26/8/2020 | 50-0 m | 1.692 | 0.154 | 1.077 | 0.000 | 0.000 | 1.231 | 0.077 | 0.000 | 0.000 |
| 26/8/2020 | 200-50 m | 2.780 | 0.024 | 3.317 | 0.073 | 0.098 | 2.732 | 0.220 | 0.000 | 0.000 |
| 26/8/2020 | 500-200 m | 0.024 | 0.012 | 0.747 | 0.048 | 0.120 | 2.988 | 0.434 | 0.000 | 0.000 |
| 26/8/2020 | 1000-500 m | 0.007 | 0.015 | 0.276 | 0.000 | 0.007 | 0.313 | 0.507 | 0.007 | 0.000 |
| 26/8/2020 | 2000-1000 m | 0.000 | 0.000 | 0.089 | 0.004 | 0.004 | 0.014 | 0.379 | 0.004 | 0.004 |
|  |  |  |  |  |  |  |  |  |  |  |
| 3/9/2020 | 50-0 m | 3.455 | 0.091 | 1.455 | 0.000 | 0.091 | 1.000 | 0.091 | 0.000 | 0.000 |
| 3/9/2020 | 200-50 m | 8.286 | 0.071 | 5.714 | 0.024 | 0.071 | 5.738 | 0.286 | 0.000 | 0.000 |
| 3/9/2020 | 500-200 m | 0.023 | 0.000 | 1.267 | 0.035 | 0.070 | 1.174 | 0.500 | 0.000 | 0.000 |
| 3/9/2020 | 1000-500 m | 0.042 | 0.007 | 0.331 | 0.014 | 0.000 | 4.000 | 0.387 | 0.000 | 0.000 |
| 3/9/2020 | 2000-1000 m | 0.010 | 0.003 | 0.136 | 0.017 | 0.000 | 0.038 | 0.259 | 0.000 | 0.000 |
|  |  |  |  |  |  |  |  |  |  |  |
| 10/9/2020 | 50-0 m | 5.083 | 0.000 | 2.167 | 0.083 | 0.000 | 1.250 | 0.083 | 0.000 | 0.000 |
| 10/9/2020 | 200-50 m | 0.300 | 0.025 | 4.850 | 0.200 | 0.075 | 1.875 | 0.450 | 0.000 | 0.000 |
| 10/9/2020 | 500-200 m | 0.000 | 0.025 | 1.519 | 0.013 | 0.038 | 1.266 | 0.684 | 0.000 | 0.000 |
| 10/9/2020 | 1000-500 m | 0.008 | 0.000 | 0.313 | 0.023 | 0.000 | 0.336 | 0.221 | 0.046 | 0.000 |
| 10/9/2020 | 2000-1000 m | 0.000 | 0.000 | 0.121 | 0.008 | 0.000 | 0.065 | 0.407 | 0.000 | 0.004 |
|  |  |  |  |  |  |  |  |  |  |  |
| 14/9/2020 | 50-0 m | 4.875 | 0.000 | 1.438 | 0.000 | 0.000 | 0.750 | 0.000 | 0.000 | 0.000 |
| 14/9/2020 | 200-50 m | 1.558 | 0.070 | 3.465 | 0.000 | 0.023 | 0.628 | 0.279 | 0.000 | 0.000 |
| 14/9/2020 | 500-200 m | 0.058 | 0.000 | 0.721 | 0.058 | 0.058 | 2.640 | 0.453 | 0.000 | 0.000 |
| 14/9/2020 | 1000-500 m | 0.015 | 0.000 | 0.207 | 0.022 | 0.000 | 0.800 | 0.281 | 0.000 | 0.000 |
| 14/9/2020 | 2000-1000 m | 0.003 | 0.000 | 0.065 | 0.007 | 0.000 | 0.010 | 0.078 | 0.007 | 0.000 |
|  |  |  |  |  |  |  |  |  |  |  |
| 16/9/2020 | 50-0 m | 3.800 | 0.000 | 1.200 | 0.000 | 0.000 | 1.100 | 0.100 | 0.000 | 0.000 |
| 16/9/2020 | 200-50 m | 1.391 | 0.000 | 3.652 | 0.065 | 0.022 | 2.522 | 0.391 | 0.000 | 0.000 |
| 16/9/2020 | 500-200 m | 0.012 | 0.000 | 0.524 | 0.060 | 0.071 | 1.702 | 0.500 | 0.000 | 0.000 |
| 16/9/2020 | 1000-500 m | 0.014 | 0.000 | 0.194 | 0.000 | 0.000 | 0.475 | 0.770 | 0.000 | 0.007 |
| 16/9/2020 | 2000-1000 m | 0.000 | 0.000 | 0.098 | 0.014 | 0.000 | 0.028 | 0.422 | 0.000 | 0.000 |
|  |  |  |  |  |  |  |  |  |  |  |
| 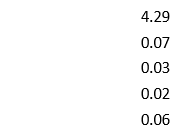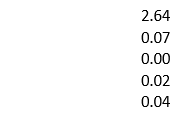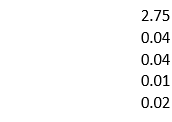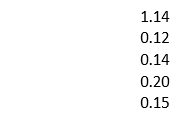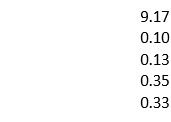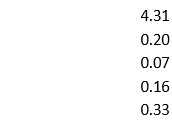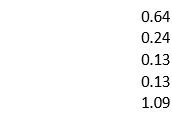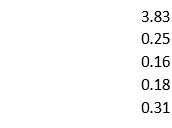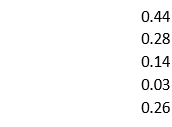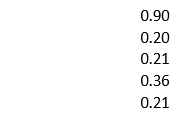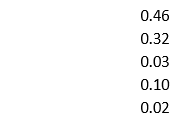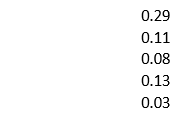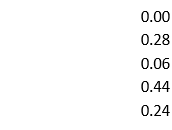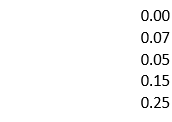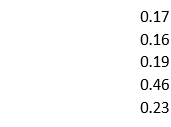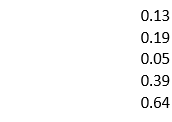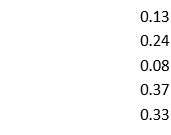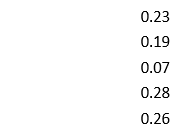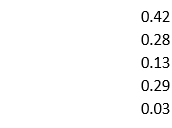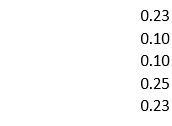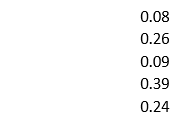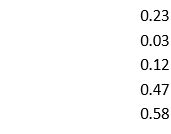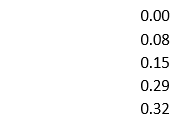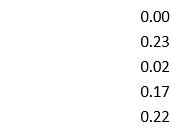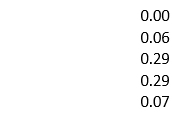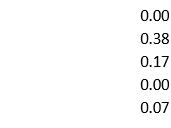24/9/2020  0-50 m  50-200 m  200-500 m  500-1000 m  1000-2000 m  Anomaly (% difference)  Anomaly (% difference)  0-50 m  50-200 m  200-500 m  500-1000 m  1000-2000 m  0-50 m  50-200 m  200-500 m  500-1000 m  1000-2000 m  0-50 m  50-200 m  200-500 m  500-1000 m  1000-2000 m  0-50 m  50-200 m  200-500 m  500-1000 m  1000-2000 m  0-50 m  50-200 m  200-500 m  500-1000 m  1000-2000 m  0-50 m  50-200 m  200-500 m  500-1000 m  1000-2000 m  0-50 m  50-200 m  200-500 m  500-1000 m  1000-2000 m  0-50 m  50-200 m  200-500 m  500-1000 m  1000-2000 m 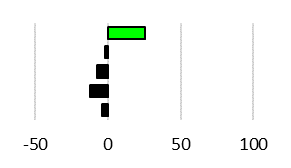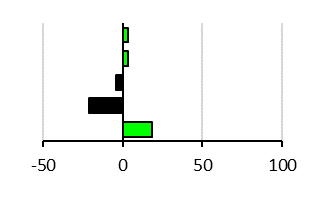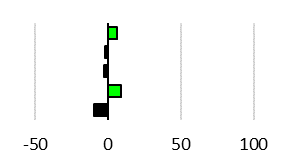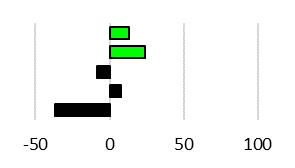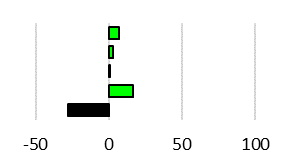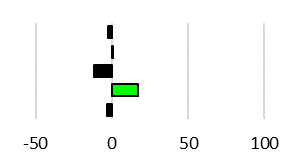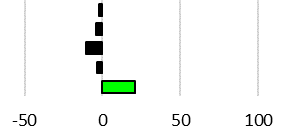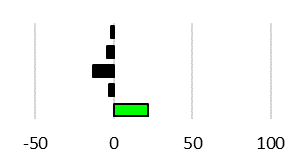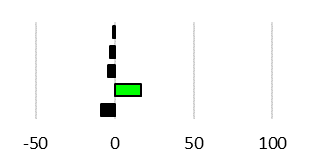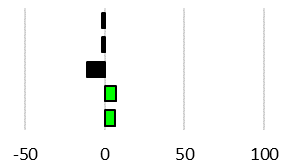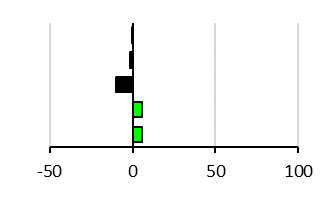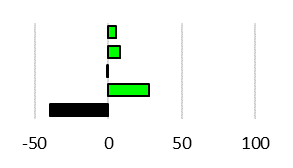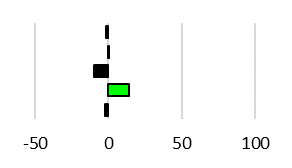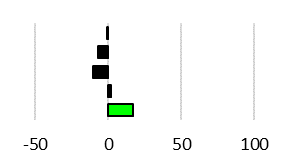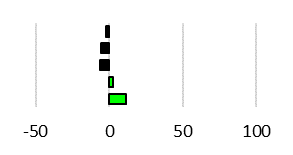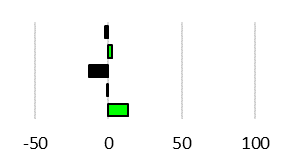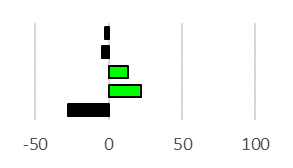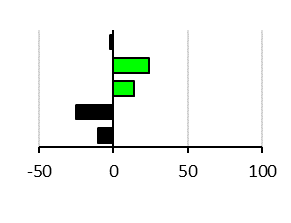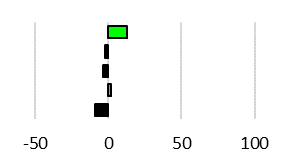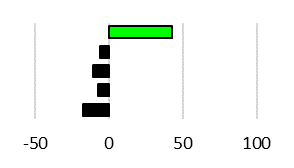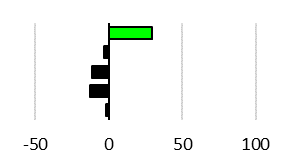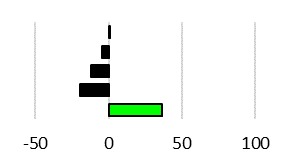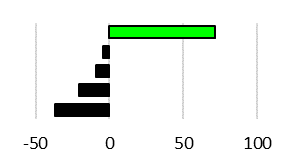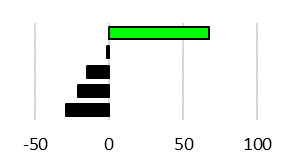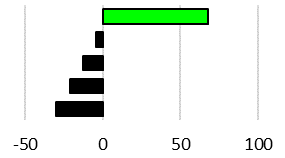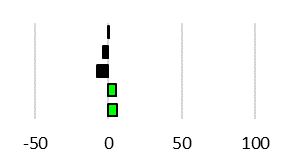 Anomaly (% difference)  Anomaly (% difference)  **16 Jul**  **23 Jul**  **27 Jul**  **17 Aug**  **18 Aug**  **26 Aug**  **3 Sep**  **10 Sep**  **14 Sep**  **16 Sep**  **24 Sep**  **25 Sep**  **14 Nov**  **28 Nov**  **5 Dec**  **21 Dec**  **26 Dec**  **2 Jan**  **7 Jan**  **14 Jan**  **21 Jan**  **29 Jan**  **4 Feb**  **18 Feb**  **7 Mar**  **12 Mar**  Anomaly (% difference)  **Fig. S1 (Supplement to Fig. 2c): Twenty-six profiles of *C. hyperboreus* CV vertical distribution (anomaly from an even distribution)**  **sampled during the MOSAiC expedition in the CAO (> 85°N) in Nov-Dec 2019, Jan-Mar 2020, Aug-Sep 2020, and in the Fram Strait**  **in July 2020. For each depth strata, the anomaly was calculated by subtracting the proportion of water sampled from the proportion of animals**  encountered, each in relation to the summed value across all depth strata (2000-0 m). | 50-0 m | 8.923 | 0.000 | 1.923 | 0.000 | 0.308 | 0.615 | 0.000 | 0.000 | 0.000 |
| 24/9/2020 | 200-50 m | 0.634 | 0.000 | 2.683 | 0.122 | 0.073 | 0.927 | 0.171 | 0.000 | 0.000 |
| 24/9/2020 | 500-200 m | 0.022 | 0.022 | 0.213 | 0.045 | 0.079 | 1.742 | 0.708 | 0.022 | 0.000 |
| 24/9/2020 | 1000-500 m | 0.000 | 0.000 | 0.151 | 0.020 | 0.013 | 1.263 | 0.224 | 0.000 | 0.013 |
| 24/9/2020 | 2000-1000 m | 0.016 | 0.003 | 0.076 | 0.013 | 0.007 | 0.289 | 0.296 | 0.007 | 0.003 |
|  |  |  |  |  |  |  |  |  |  |  |
| 25/9/2020 | 50-0 m | 15.000 | 0.071 | 0.929 | 0.000 | 0.071 | 0.429 | 0.000 | 0.000 | 0.000 |
| 25/9/2020 | 200-50 m | 0.895 | 0.000 | 3.447 | 0.158 | 0.053 | 0.711 | 0.237 | 0.000 | 0.000 |
| 25/9/2020 | 500-200 m | 0.068 | 0.000 | 0.176 | 0.068 | 0.216 | 1.932 | 0.622 | 0.000 | 0.000 |
| 25/9/2020 | 1000-500 m | 0.015 | 0.007 | 0.213 | 0.015 | 0.007 | 0.338 | 0.272 | 0.015 | 0.000 |
| 25/9/2020 | 2000-1000 m | 0.000 | 0.000 | 0.078 | 0.004 | 0.019 | 0.033 | 0.316 | 0.007 | 0.000 |


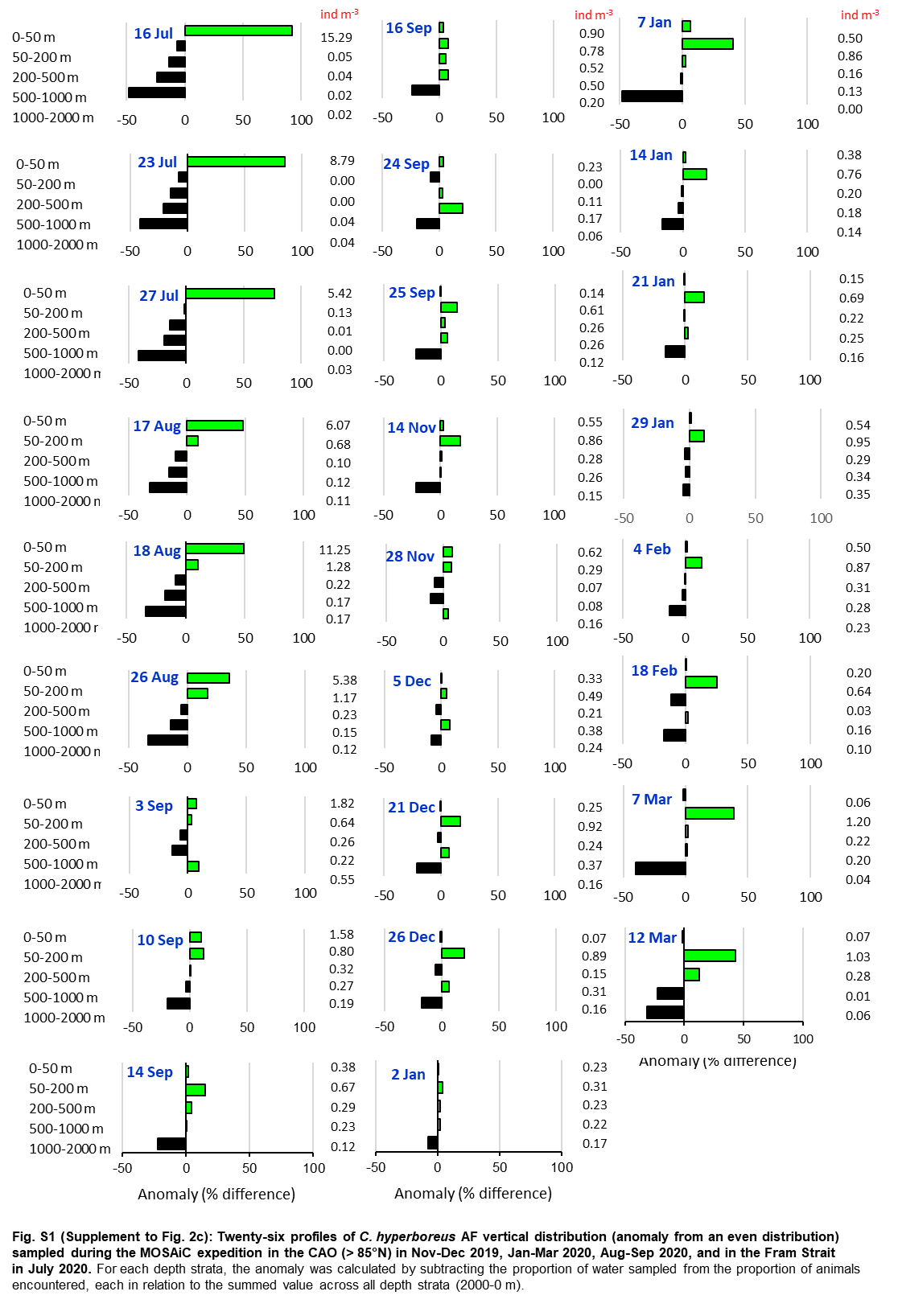


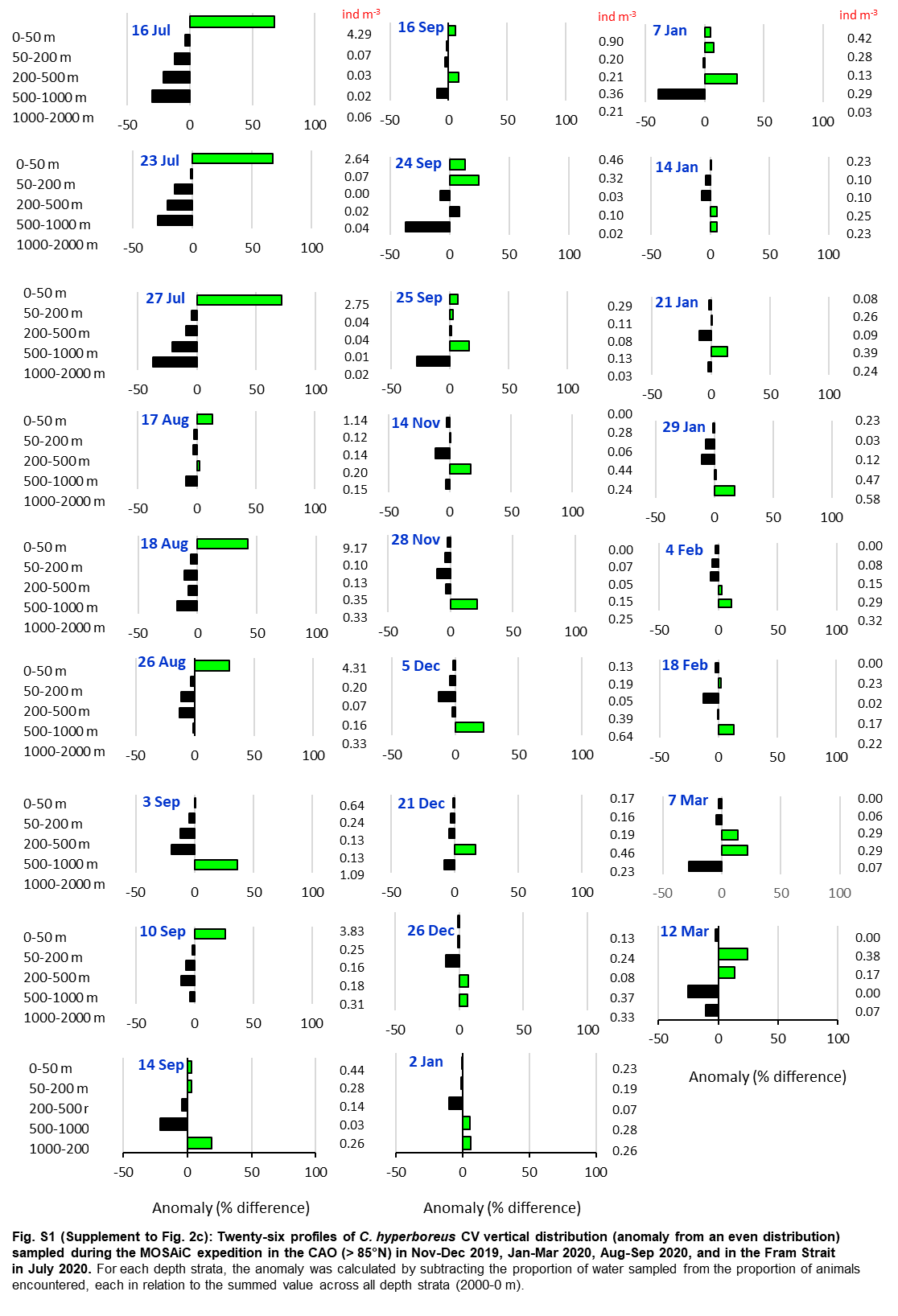


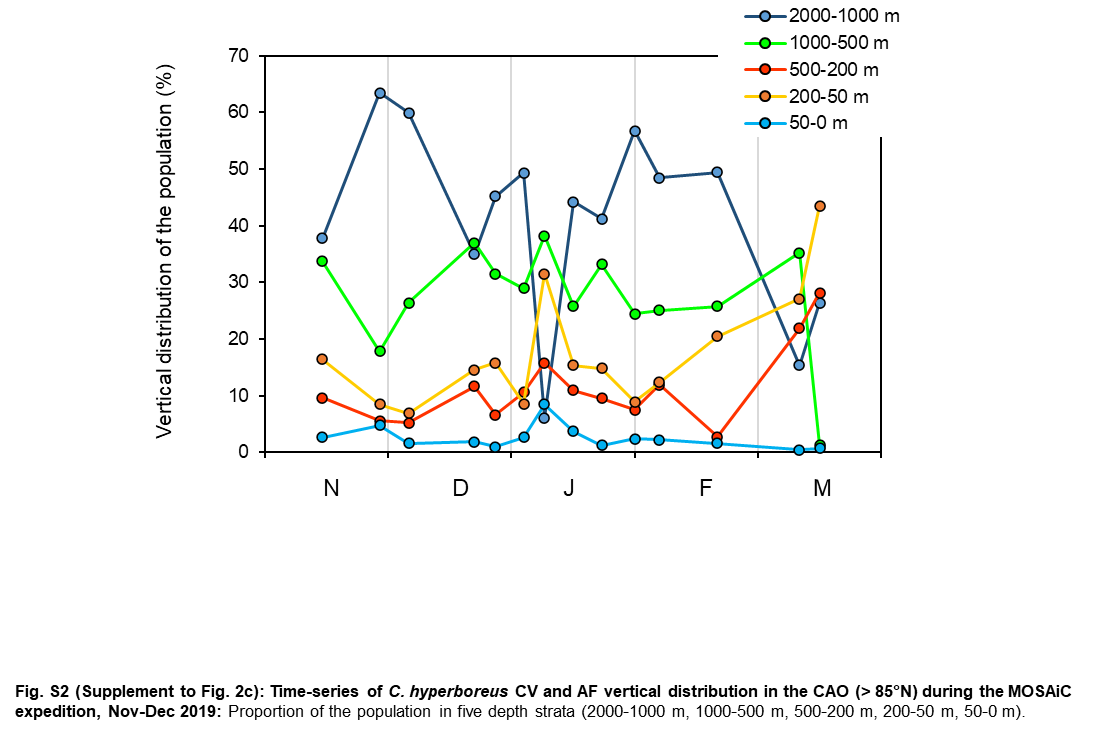


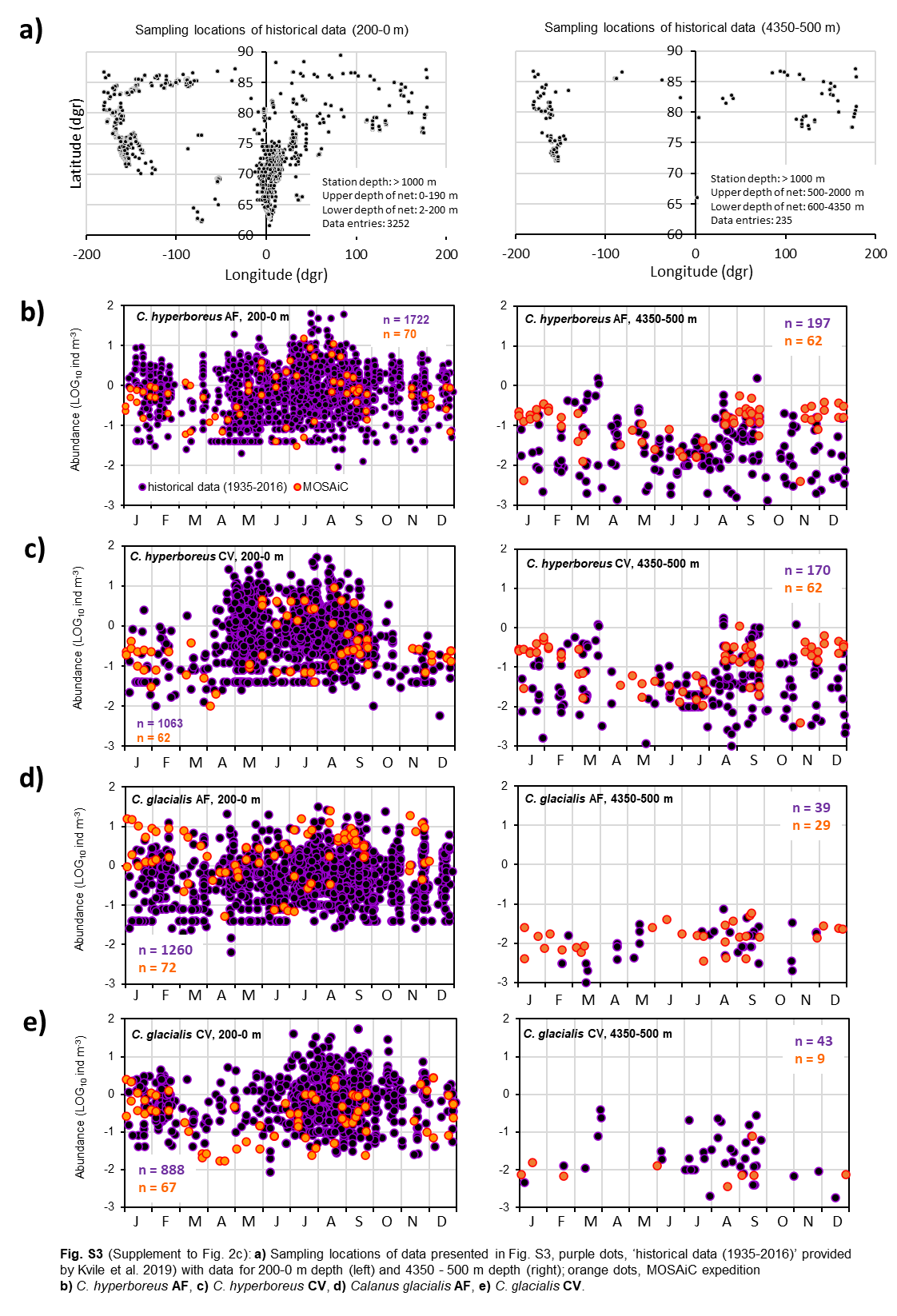


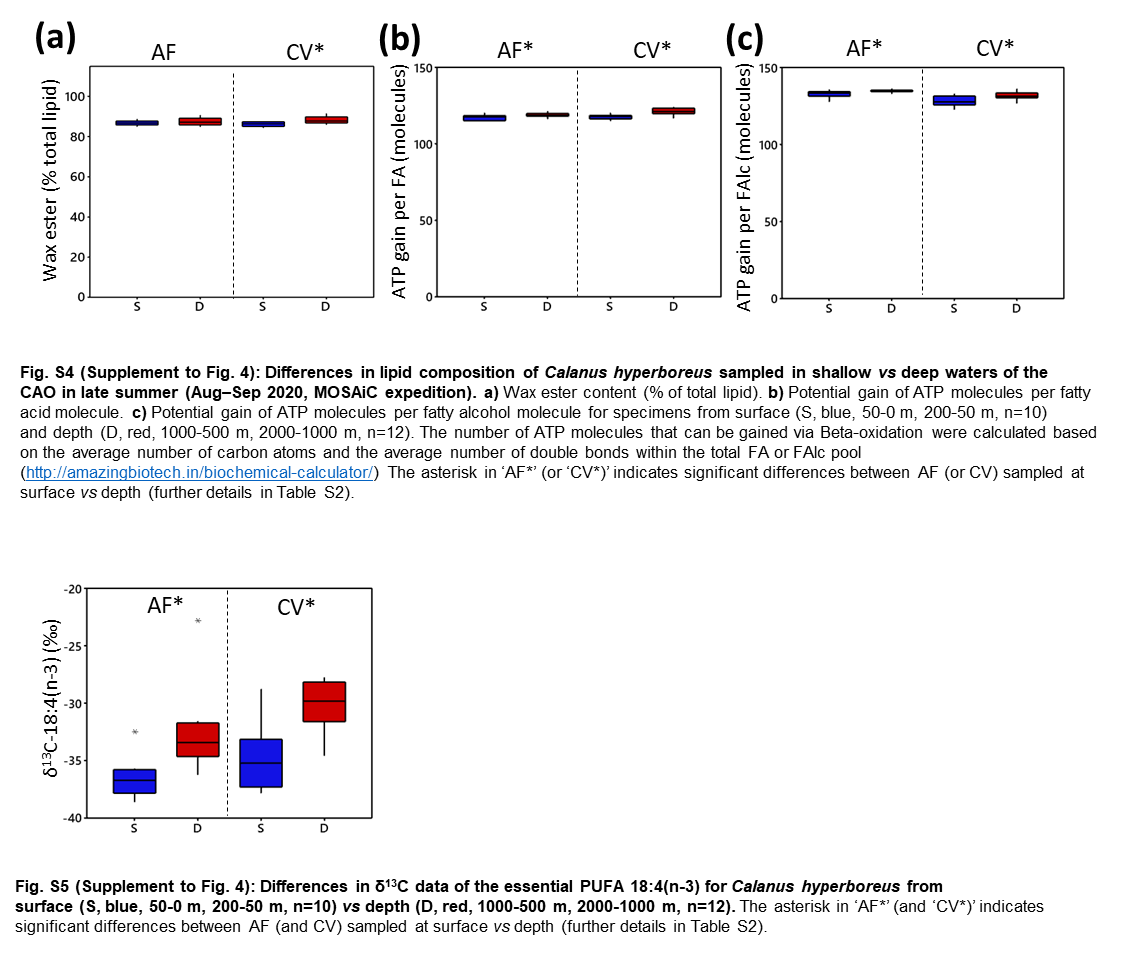


**Fig. S6**

**Developmental stage composition of the *Calanus hyperboreus* population in the CAO (≥ 85°N, MOSAiC expedition) in Nov 2019-Mar 2020 and Aug-Sep 2020.**

The eggs were only identified to group level (copepods) and the nauplii to genus level (*Calanus*), but likely derived to a large extend from *Calanus hyperboreus* due to their unique wintertime reproduction compared to the other *Calanus* species.
