# Supplementary material for: Seasonal vertical migration of large polar copepods reinterpreted as a dispersal mechanism throughout the water column
Source: Commun Earth Environ. 2025 Jun 4;6(1):431. doi: 10.1038/s43247-025-02389-9 (PMC12137130; doi:10.1038/s43247-025-02389-9)
Supplement: Supplementary file 3 — Reporting Summary [file 43247_2025_2389_MOESM3_ESM.pdf]

nature portfolio

Corresponding author(s): Katrin Schmidt

Last updated by author(s): May 9, 2025

Reporting Summary

Nature Portfolio wishes to improve the reproducibility of the work that we publish. This form provides structure for consistency and transparency in reporting. For further information on Nature Portfolio policies, see our Editorial Policies and the Editorial Policy Checklist.

Statistics

For all statistical analyses, confirm that the following items are present in the figure legend, table legend, main text, or Methods section.

n/a	Confirmed	
☐	☒	The exact sample size (*n*) for each experimental group/condition, given as a discrete number and unit of measurement
☐	☒	A statement on whether measurements were taken from distinct samples or whether the same sample was measured repeatedly
☐	☒	The statistical test(s) used AND whether they are one- or two-sided<br>*Only common tests should be described solely by name; describe more complex techniques in the Methods section.*
☒	☐	A description of all covariates tested
☐	☒	A description of any assumptions or corrections, such as tests of normality and adjustment for multiple comparisons
☐	☒	A full description of the statistical parameters including central tendency (e.g. means) or other basic estimates (e.g. regression coefficient) AND variation (e.g. standard deviation) or associated estimates of uncertainty (e.g. confidence intervals)
☐	☒	For null hypothesis testing, the test statistic (e.g. *F*, *t*, *r*) with confidence intervals, effect sizes, degrees of freedom and *P* value noted<br>*Give P values as exact values whenever suitable.*
☒	☐	For Bayesian analysis, information on the choice of priors and Markov chain Monte Carlo settings
☒	☐	For hierarchical and complex designs, identification of the appropriate level for tests and full reporting of outcomes
☒	☐	Estimates of effect sizes (e.g. Cohen's *d*, Pearson's *r*), indicating how they were calculated

Our web collection on statistics for biologists contains articles on many of the points above.

Software and code

Policy information about availability of computer code

Data collection	no software was used to collect the data
Data analysis	Minitab v17

For manuscripts utilizing custom algorithms or software that are central to the research but not yet described in published literature, software must be made available to editors and reviewers. We strongly encourage code deposition in a community repository (e.g. GitHub). See the Nature Portfolio guidelines for submitting code & software for further information.

Data

Policy information about availability of data

All manuscripts must include a data availability statement. This statement should provide the following information, where applicable:

- Accession codes, unique identifiers, or web links for publicly available datasets
- A description of any restrictions on data availability
- For clinical datasets or third party data, please ensure that the statement adheres to our policy

Provide your data availability statement here.

Human research participants

Policy information about studies involving human research participants and Sex and Gender in Research.

Reporting on sex and gender	N/A
Population characteristics	N/A
Recruitment	N/A
Ethics oversight	N/A

Note that full information on the approval of the study protocol must also be provided in the manuscript.

Field-specific reporting

Please select the one below that is the best fit for your research. If you are not sure, read the appropriate sections before making your selection.

☐ Life sciences ☐ Behavioural & social sciences ☒ Ecological, evolutionary & environmental sciences

For a reference copy of the document with all sections, see nature.com/documents/nr-reporting-summary-flat.pdf

Ecological, evolutionary & environmental sciences study design

All studies must disclose on these points even when the disclosure is negative.

Study description	Zooplankton field sampling the the Central Arctic Ocean for taxonomical and life stage identification, and the analysis of body conditions and feeding history. Sampling effort was restricted by the ship time. There were 26 sampling events over the duration of one year (Nov 2019-Sep 2020)
Research sample	The study focussed primarily on the copepods Calanus hyperboreus and Calanus glacialis (subadults and adults) with minor emphasis on their potential predators (other copepods, cnidaria, chaetognaths, amphipods ect.)
Sampling strategy	Per sampling event, one vertical MultiNet cast was taken with five nets, sampling 2000-1000 m, 1000-500 m, 500-200 m, 200-50 m, 50-0 m. There was no ship-time for replicate sampling.
Data collection	Taxonomic analysis of the samples was carried out at the Alfred Wegener Insititut Bremerhaven (Germany). Biochemical analysis of the samples was carried out at the University of Plymouth (UK), AWI Bremerhaven (Germany) and University of La Rochelle (France). Die biochemical data include total lipid content, wax ester content, fatty acid composition, sterol composition, abundance of highly-branched isoprenoids, stable isotope composition and compound-specific stable isotopes.
Timing and spatial scale	Samples were taken during the MOSAiC expedition (Nov 2019-Sep 2020), with two major sampling events: Nov 2019-Mar 2020 (14 sampling events); and Jul 2020-August 2020 (12 sampling events). The first period represents Arctic winter and the second Arctic summer. Samples were taken in the Amundsen Basin, Nansen Basin and Fram Strait.
Data exclusions	No data were excluded from the analysis.
Reproducibility	This was a field sampling program, not an experiment. However, we compared the vertical distribution data with those collected previously during ice drift expeditions in the Central Arctic Ocean in 1950-1956 (Severny Polyus) and 1997/1998 (SHEBA).
Randomization	For taxonomic analysis either the whole sample was analysed (rare species) or subsamples were taken via a Folsom-splitter (Plankton Splitter), which is a common practice. For biochemical analysis, large, rare zooplankton were analysed in batches of three at the minimum; smaller, common zooplankton was analysed in batches of 10-50 specimens, according to availability from net sampling.
Blinding	No Blinding was carried out.

Did the study involve field work? ☒ Yes ☐ No

Field work, collection and transport

Field conditions	Water temperatures varied between -1.7 to +2 dgr C. Winter samples were taken during Polar night and summer samples during Polar Day.

Location	Samples were collected primarily in the Amundsen Basin (> 85 dgr N), with some in the Nansen Basin and Fram Strait.
Access & import/export	Zooplankton samples were collected in international waters. None of the species is considered endangered. On-board, samples for taxonomical analysis were preserved in Formalin, samples for biochemical analysis were frozen at -80 dgr C. The initial landing was at the AWI (Bremerhaven). Taxonomical samples were analysed at the AWI. Samples for biochemical analysis were freeze-dried at the AWI and then posted to UK via DHL. No permission was required as the samples included invertebrates and the posting happened before BREXIT regulations.
Disturbance	The overall numbers of animals removed from the field was very low compared to the natural abundances that occur in the high Arctic.

Reporting for specific materials, systems and methods

We require information from authors about some types of materials, experimental systems and methods used in many studies. Here, indicate whether each material, system or method listed is relevant to your study. If you are not sure if a list item applies to your research, read the appropriate section before selecting a response.

Materials & experimental systems

n/a	Involved in the study
☒	☐ Antibodies
☒	☐ Eukaryotic cell lines
☒	☐ Palaeontology and archaeology
☒	☐ Animals and other organisms
☒	☐ Clinical data
☒	☐ Dual use research of concern

Methods

n/a	Involved in the study
☒	☐ ChIP-seq
☒	☐ Flow cytometry
☒	☐ MRI-based neuroimaging
